# Design and application of a novel PNA probe for the detection at single cell level of JAK2^V617F^ mutation in Myeloproliferative Neoplasms

**DOI:** 10.1186/1471-2407-13-348

**Published:** 2013-07-18

**Authors:** Enrico Bracco, Valentina Rosso, Anna Serra, Francesca Carnuccio, Valentina Gaidano, Paolo Nicoli, Pellegrino Musto, Giuseppe Saglio, Francesco Frassoni, Daniela Cilloni

**Affiliations:** 1 Division of Hematology and Internal Medicine, Department of Oncology, University of Turin, Turin, Italy; 2 Department of Onco-Hematology, Centro di Riferimento Oncologico della Basilicata (CROB), IRCCS, Rionero in Vulture, Italy; 3 Stem Cell and Cellular Therapy Laboratory, G. Gaslini Institute, Genova, Italy

**Keywords:** JAK2, Myeloproliferative neoplasms, PNA

## Abstract

**Background:**

Mutation(s) of the *JAK2* gene (V617F) has been described in a significant proportion of Philadelphia negative Myeloproliferative Neoplasms (MPN) patients and its detection is now a cornerstone in the diagnostic algorithm.

**Methods:**

We developed a novel assay based on peptide nucleic acid (PNA) technology coupled to immuno-fluorescence microscopy (PNA-FISH) for the specific detection at a single cell level of *JAK2*-mutation thus improving both the diagnostic resolution and the study of clonal prevalence.

**Results:**

Using this assay we found a percentage of mutated CD34+ cells ranging from 40% to 100% in Polycythemia Vera patients, from 15% to 80% in Essential Thrombocythemia and from 25% to 100% in Primary Myelofibrosis. This method allows to distinguish, with a high degree of specificity, at single cell level, between CD34+ progenitor stem cells harbouring the mutated or the wild type form of *JAK2* in NPM patients.

**Conclusions:**

This method allows to identify multiple gene abnormalities which will be of paramount relevance to understand the pathophysiology and the evolution of any type of cancer.

## Background

**Figure 1:**
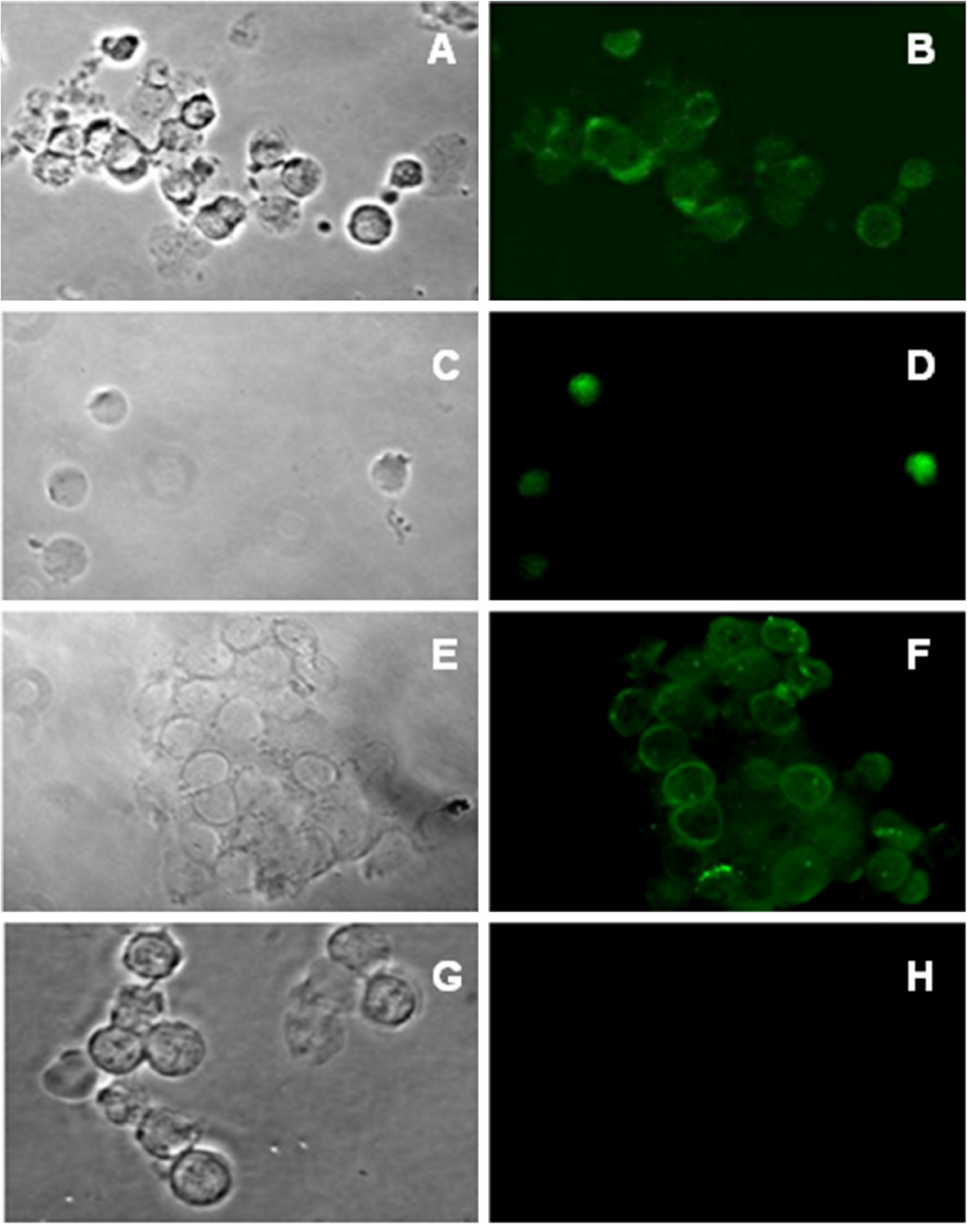
**Detection of JAK2 mutation by PNA.** Detection of *JAK2*^*V617F*^ mutation by PNA (green signal) in CD34+ cells enriched from patients affected by Essential Thrombocytemia (ET), **(A, B)**, Primary myelofibrosis (PMF) **(C, D)** Polycytemia Vera (PV) **(E, F)**. Negative control **(G, H)** is represented by a non JAK2 mutated MPN patient. No specific PNA green signal can be detected in the absence of *JAK2*^*V617F*^ mutation.

Ph-negative MPNs include Essential Thrombocytemia, Polycythemia Vera and Primary Myelofibrosis. They share a common molecular signature represented by the mutation of *JAK2*[1-4]. The detection *JAK2*^*V617*^ is included in the diagnostic criteria and specific JAK2 inhibitors have been recently approved for the treatment of these patients [1]. MPNs are currently considered stem cell-related disorders of monoclonal origin although the presence of different co-existing subclones cannot be ruled out. Interestingly, in patients bearing *JAK2*^*V617F*^ within the CD34+ compartment a mosaicism of cells harbouring the J*AK2*^*V617F*^ can be detected alongside with the wild type counterparts, as elegantly reported by Scott and colleagues [5]. Nevertheless current approaches do not discriminate these two populations or directly quantify them in a easy and affordable format. In particular, the available methodology forecasts the use of colony formation assays followed by capillary electrophoresis sequencing [5,6]. Overall, this method owns some pitfalls which are primarily due to: i) time consuming sample processing and ii) relatively low abundance of DNA isolated from colonies, iii) culture conditions (i.e. Epo +/−) may alter the proportion of colonies bearing the mutation. On the other hand, the method most frequently used for measuring the distribution of cell populations is indirect and based on *JAK2* sequencing. The *JAK2*^*V617F*^ allele-burden is usually estimated by allele specific polymerase chain reaction (PCR). Although it is a sensitive assay, this is performed on the whole ‘white’ myeloid differentiated cell population (e.g. granulocytes) and for this reason it may be biased by ‘dilution effects’ on sample. For all this reasons, the chance of distinguishing the *JAK2V617F* at the single-cell level still represents a challenge, both in the diagnostic and research field. The PNA is a synthetic nucleic acid analogue in which the negatively charged sugar phosphate backbone is replaced by a neutral pseudo-peptide backbone [7]. Due to its (i) high degree of sequence selectivity, (ii) discrimination ability in binding to complementary DNA or RNA, and (iii) increased stability (relative to non-synthetic nucleic acids) and (iiii) low cost, PNA probes do have tremendous potential for therapeutic application as well as diagnostic and research use, especially when a highly specific matching is needed. The physical properties of PNA endow them with specific advantages over standard oligonucleotides probes: they are less polar than (natural) nucleic acids and -as a consequence- PNA/DNA heteroduplexes are thermodynamically favoured when compared to the DNA/DNA double helix [8]. When very short PNA are used, this greater specificity allows the PNA/DNA heteroduplex to become thermodynamically unstable even when a single base-pair mismatch occurs [9]. Taking advantage of these unique PNA features we set-up a fluorescently-labelled PNA probe, coupled to FISH technology, to identify the presence of J*AK2*^*V617F*^ at the single-cell level. This method allows to distinguish between CD34+ progenitor stem cells harbouring the mutated or wild type form of *JAK2* (Figure 1).

In this article we report a method characterized by a high degree of specificity and sensitivity which allows to identify at a single cell level the presence of JAK2^V617F^ mutation.

## Results and discussion

**Figure 2:**
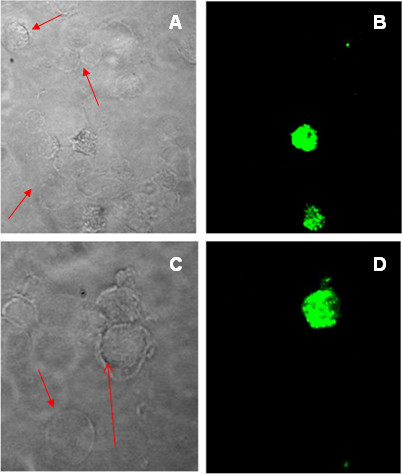
**PNA distinguishes between CD34+ cells with and without JAK2 mutation in PV patient.** Detection of *JAK2*^*V617F*^ mutation by PNA (green signal) in some CD34+ cells enriched from patients affected by Polycytemia Vera **(B, D)**. Red Arrows in panels **(A, C)** indicate CD34+ cells without the *JAK2* mutation. The percentage of PNA positive and negative cells are variable among patients and in different diseases.

CD34+ cells from *JAK2*^*V617F*^ positive patients (affected by ET, PV and PMF) displayed an heterogeneous staining pattern when probed with the *JAK2*^*V617F*^/PNA. Indeed, in a single patient some CD34+ cells are clearly positive for *JAK2*^*V617F*^/PNA-fluorescent staining, while others are negative (Figure 2). The analysis revealed that among *JAK2*^*V617F*^ PV patients the distribution pattern is fairly similar to that reported by Scott *et al.*[5] with a rather wide variability occurring among patients. We found a percentage of mutated CD34+ cells ranging from 40% to 100% in PV patients, from 15% to 80% in ET and from 25% to 100% in PMF. These findings are in agreement with previous data reporting that a variable proportion of progenitors from patients affected by *JAK2*^*V617F*^ positive PV are capable of generating *JAK2*^*V617F*^ negative colonies [5]. In addition these data indicate that fluorescinated *JAK2*^*V617F*^/PNA probe displays a very high specificity towards a single base-pair mismatch.

Interestingly, when evaluating the presence of *JAK2*^*V617F*^positive cells collected from *JAK2* wild type subjects defined by sequencing and by Q-PCR we identified a small percentage of cells positive for the *JAK2*^*V617F*^/PNA staining not exceeding 3% of the CD34+ cell population indicating a high level of sensitivity of the procedure. Interestingly, this apply only to patients with PV but not with PMF and ET. Importantly, the lack of positivity detected in CD34+ cells from 20 healthy subjects demonstrates a high specificity of this method. We conclude that the *JAK2*^*V617F*^/PNA-FISH method displays high specificity and reliability in discriminating cell subpopulations harbouring the *JAK2*^*V617F*^ mutation. In addition, it allows to analyze the CD34+ population at the single cell level, avoiding the time consuming analysis of hematopoietic colonies. The fact that our results are in keeping with the data reported by Scott et al [5] corroborate the robustness of the technique although we think that the proposed approach is much easier and free from variability related to colony growth conditions [5,6]. This approach allows to monitor longitudinally the evolution of a defined cell population over time in MPNs.

## Conclusions

This study presents a novel PNA-FISH protocol which allows to characterize the CD34+ compartment in patients with MPNs. These data could improve the knowledge on the pathophysiology of MPNs and will improve both the diagnostic and discovery tools. In addition, our approach allows the identification of specific genetic (or gene) abnormalities in any type of cancer. This is very relevant since in the great majority of neoplasm a technique equivalent to the *in vitro* growth of progenitor cells is unavailable.

## Methods

### Patients

Local ethic committee San Luigi Gonzaga, Piedmont Region, approved the study (approval number 203). After informed consent BM aspirates were obtained from 24 PV patients (5 of them were selected for the absence of *JAK2*^*V617F*^ ), 13 PMF (3 of them were *JAK2* wild type) and 6 ET patients (2 of them were wild type). In addition 20 BM samples were collected from healthy donors and used as control.

### CD34+ enrichment

CD34+ cells were enriched by magnetic cell sorting (MACS) (Miltenyi Biotec, Bergisch Gladbach, Germany) according to the manufacturer’s protocol.

### Capillary Sanger sequence method for the detection of JAK2^V617F^

Detection of *JAK2*^*V617F*^ was performed by capillary Sanger Sequence method. JAK2^V617F^ mutation was amplified using primers described by Baxter and colleagues [4] and analyzed by sequencing with BigDye terminator v3.1 (Applied Biosystem, Foster City, California CA) and capillary electrophoresis on ABI PRISM 3130XL (Applied Biosystem, Foster City, California CA). The sensitivity of this method was previously estimated by serial dilutions experiments to be 10%.

### Quantitative PCR

Q-PCR was performed by making use of JAK2 MutaQuant kit (Ipsogen, Marseille, France) based on Taqman technology according to the manufactures’ procedure. The signal is measured on a standard curve. The sensitivity of this method was estimate to be 0.1%.

### Design and sequence of JAK2^V617F^ PNA probe

PNA probe, designed on the human JAK2 cDNA (acc. # NM_004972), encompasses a very short sequence (12 nucleotides) just over the codon 617 (bp 1849 when referring to the coding sequence) responsible for the V- > F mutation. The single nucleotide mismatch falls just in the middle of the sequence. The probe has been further tagged by fluorescinated dye at its amino-terminus. The sequence is as follow: Alexa488-OO-GTATGTTTCTGT-Lys.

### Protocol for JAK2V617F detection using a specific PNA probe

1. Fiveml cell culture medium were prepared usingrpmI medium enriched with 20% Fetal Bovine Serum (FBS). Oneml of fresh marrow blood was put in culture. CD34+ cells were enriched from bone marrow aspirates by magnetic cell sorting (MACS; Miltenyi Biotec, Bergisch Gladbach, Germany) following the manufacture’s instruction.

2. CD34+ were incubated over night (ON) at 37 C in 5% CO_2_ atmosphere.

3. Cells were harvest by centrifugation (1500 rpm for 7 minutes).

4. Supernatant was carefully removed.

5. Fiveml (Phosphate Buffer Saline) PBS was added and cells re-suspended.

6. Cells were harvested by centrifugation as in step 3.

7. Pellet was re-suspend in 10 ml of 75 mM KCl, and incubated at 37°C for 20 minutes.

Note: Mix carefully by vortexing.

8. Cells were harvest by centrifugation as previously described in step 3.

9. Supernatant was removed.

10. Threeml of a freshly made Methanol ice cold:acetic acid (3:1) solution were added.

Note: Prepare just before use. It is very important adding the methanol:acetic acid very carefully and slowly.

11. Cells were harvest by centrifugation.

12. Supernatant was removed

13. Steps 10, 11, 12 were repeated four times.

14. Cells re-suspended in acetic acid solution and Cytospun on slides (500 rpm for 10 minutes; at least 10 [5] cells per slide).

15. Slides were immersed in 2X SSC (Sodium Citrate Solution, Invitrogen) solution at 37°C for 30 minutes.

16. Slides were dehydrated in cold ethanol series (for 2 minutes each in 70%, 80%, 90%).

Note: Avoid to drying slides during serial passages.

17. Slides were dried on air.

18. Threeml PNA probe were added to each slides (final concentration 200 nM).

19. Slides were covered with a coverslip and seal with glue (Rubber Cement, Fixogum, LK-071° KREATECH diagnostics).

Note: Be careful: after adding PNA maintain the slides in dark to avoid fluorescence bleaching.

20. DNA was denatured by incubating the slide for 2 minutes at 82°C and then incubate ON at 37°C.

21. Coverslips were removed.

22. Slides were washed I the in 0.5X SSC for 5 minutes at 65°C.

23. Slides were washed the in PBS 1X at room temperature for 30 seconds.

24. Step 23 was repeated (× 3).

25. Coverslip was used to allow the solution to spread evenly under the coverslip. Avoid air bubbles.

Note: Slides were placed in the dark at 4°C for at least couple of hours.

26. Stained slide were analyzed by using an fluorescence microscope with appropriate filters.

27. Slides were stored the in the dark at 4°C up to few weeks.

## Abbreviations

BM: Bone marrow; EPO: Erythropoietin; ET: Essential thrombocythemia; FISH: Fluorescence in situ hybridization; JAK2: Janus kinase 2; MACS: Magnatic cell sorting; MPN: Myeloproliferative neoplasms; ON: Over night; Q-PCR: Quantitative polymerase chain reaction; PMF: Primary myelofibrosis; PNA: Peptide nucleic acid; PV: Polycythemia vera.

## Competing interests

The authors have no competing financial interests.

## Authors’ contributions

EB designed the PNA, supervised the experiments. VR set up and performed the PNA experiments. AS and FC performed JAK2 analysis by sequences and Q-PCR. PM and PN provided samples and analyzed clinical data. GS provided final approval. FF analyzed the data and wrote the manuscript. DC designed the study, analyzed the data and wrote the manuscript. All authors’ read and approved the final manuscript.

## Pre-publication history

The pre-publication history for this paper can be accessed here:

http://www.biomedcentral.com/1471-2407/13/348/prepub

## References

[B1] Tefferi A Veinchenker W Myeloproliferative neoplasms: molecular pathophysiology, essential clinical understanding, and treatment strategies J Clin Oncol 2011 29 573 582 10.1200/JCO.2010.29.8711 21220604

[B2] Kralovics R Passamonti F Buser AS Teo SS Tiedt R Passweg JR A gain-of-function mutation of JAK2 in myeloproliferative disorders N Engl J Med 2005 352 1779 1790 10.1056/NEJMoa051113 15858187

[B3] Levine RL Wadleigh M Cools J Ebert BL Wernig G Huntly Activating mutation in the tyrosine kinase JAK2 in polycythemia vera, essential thrombocythemia, and myeloid metaplasia with myelofibrosis Cancer Cell 2005 7 387 397 10.1016/j.ccr.2005.03.023 15837627

[B4] Baxter EJ Scott LM Campbell PJ East C Fourouclas N Swanton S Acquired mutation of the tyrosine kinase JAK2 in human myeloproliferative disorders Lancet 2005 365 1054 1061 15781101 10.1016/S0140-6736(05)71142-9

[B5] Scott LM Scott MA Campbell PJ Green AR Progenitors homozygous for the V617F mutation occur in most patients with polycythemia vera, but not essential thrombocythemia Blood 2006 108 2435 2437 10.1182/blood-2006-04-018259 16772604

[B6] Jamieson CHM Gotlib J Durocher JA Chao MP Mariappan MR Lay M The JAK2 V617F mutation occurs in hematopoietic stem cells in polycythemia vera and predisposes toward erythroid differentiation Proc Natl Acad Sci USA 2006 103 6224 6229 10.1073/pnas.0601462103 16603627 PMC1434515

[B7] Egholm M Buchardt O Christensen L Behrens C Freier SM Driver DA PNA hybridizes to complementary oligonucleotides obeying the Watson-Crick hydrogen-bonding rules Nature 1993 365 566 568 10.1038/365566a0 7692304

[B8] Sugimoto N Yamamoto K Satoh N Positional effect of single bulge nucleotide on PNA(peptide nucleic acid)/DNA hybrid stability Nucleic Acids Symp Ser 1999 42 95 96 10.1093/nass/42.1.95 10780396

[B9] Sugimoto N Satoh N Yamamoto K Comparison of thermodynamic stabilities between PNA (peptide nucleic acid)/DNA hybrid duplexes and DNA/DNA duplexes Nucleic Acids Symp Ser 1999 42 93 94 10.1093/nass/42.1.93 10780395

